# A new species of Epeorus (Caucasiron) (Ephemeroptera, Heptageniidae) from Azerbaijan and Iran

**DOI:** 10.3897/zookeys.1068.70717

**Published:** 2021-11-02

**Authors:** Ľuboš Hrivniak, Pavel Sroka, Jindřiška Bojková, Roman J. Godunko, Peter Manko

**Affiliations:** 1 Biology Centre of the Czech Academy of Sciences, Institute of Entomology, Branišovská 31, 37005 České Budějovice, Czech Republic Biology Centre of the Czech Academy of Sciences, Institute of Entomology České Budějovice Czech Republic; 2 Department of Botany and Zoology, Masaryk University, Kotlářská 2, 61137 Brno, Czech Republic Masaryk University Brno Czech Republic; 3 Department of Invertebrate Zoology and Hydrobiology, University of Łódź, Banacha 12/16, 90237 Łódź, Poland University of Łódź Łódź Poland; 4 Department of Ecology, Faculty of Humanities and Natural Sciences, University of Prešov, 17. novembra 1, 08116 Prešov, Slovakia University of Prešov Prešov Slovakia

**Keywords:** Caucasus, mayflies, molecular species delimitation, taxonomy

## Abstract

A new species, Epeorus (Caucasiron) hyrcanicus**sp. nov.**, is described based on larval morphology and molecular data (COI) containing sequences from all Caucasian *Caucasiron* species described to date. The species is distributed in the Hyrcanian forest of southeastern Azerbaijan and northwestern Iran. Based on our wide-range sampling, the new species is likely endemic to this area. The most pronounced larval morphological diagnostic characters are the coloration pattern of abdominal sterna (a pair of oblique stripes and stripe-like medio-lateral maculae) and terga (triangular medial maculae), poorly developed projection of the costal margin of gill plates III, presence of hair-like setae on the surface of abdominal terga, and relatively wide shape of gill plates VII (in natural position from ventral view). The diagnostic characters are compared to related species, and primary information to habitat is provided.

## Introduction

*Epeorus* Eaton, 1881 s.l. is one of the most diverse mayfly genera in the Caucasus region. Except for a single representative of *Epeorus* s.str., Epeorus (Epeorus) zaitzevi Tshernova 1981, all *Epeorus* species distributed in the region belong to the subgenusCaucasiron Kluge, 1997 (hereinafter *Caucasiron*).

The global distribution of *Caucasiron* includes the eastern Mediterranean islands (Samos and Cyprus), Turkey, the Caucasus, Iraq, Iran, Central Asia (Kazakhstan, Tajikistan, Nepal, and India), and south-western China (Guizhou province) (e.g. Chen 2010; Bojková et al. 2018; Hrivniak et al. 2020a; Khudhur and Sroka 2021). The highest diversity is known in the Caucasus and adjacent areas (including Samos Island), where 15 species have been described to date (Hrivniak et al. 2020a). Central Asia and south-western China are each inhabited by a single species, altogether numbering 17 confirmed *Caucasiron* species worldwide.

Currently, the following species are known from the Caucasus and adjacent areas (Hrivniak et al. 2020a, 2020b): E. (C.) caucasicus (Tshernova, 1938), E. (C.) znojkoi (Tshernova, 1938), E. (C.) nigripilosus (Sinitshenkova, 1976), E. (C.) magnus (Braasch, 1978), E. (C.) alpestris (Braasch, 1979), E. (C.) soldani (Braasch, 1979), E. (C.) sinitshenkovae (Braasch & Zimmerman, 1979), E. (C.) iranicus (Braasch & Soldán, 1979), E. (C.) longimaculatus (Braasch, 1980), E. (C.) insularis (Braasch, 1983), E. (C.) bicolliculatus Hrivniak, 2017, E. (C.) turcicus Hrivniak, Türkmen & Kazancı, 2019, E. (C.) alborzicus Hrivniak & Sroka, 2020, E. (C.) shargi Hrivniak & Sroka, 2020, and E. (C.) zagrosicus Hrivniak & Sroka, 2020. However, a recent molecular study of Caucasian *Caucasiron* by Hrivniak et al. (2020c) revealed several additional evolutionary lineages, indicating that species diversity of *Caucasiron* in the Caucasus could be higher. The morphology of these lineages was not examined in detail, and these taxa were, therefore, left without formal description.

In this study, we provide a detailed morphological investigation and description of the lineage labelled as *Caucasiron* sp. 3 by Hrivniak et al. (2020c).

**Figure 1.**
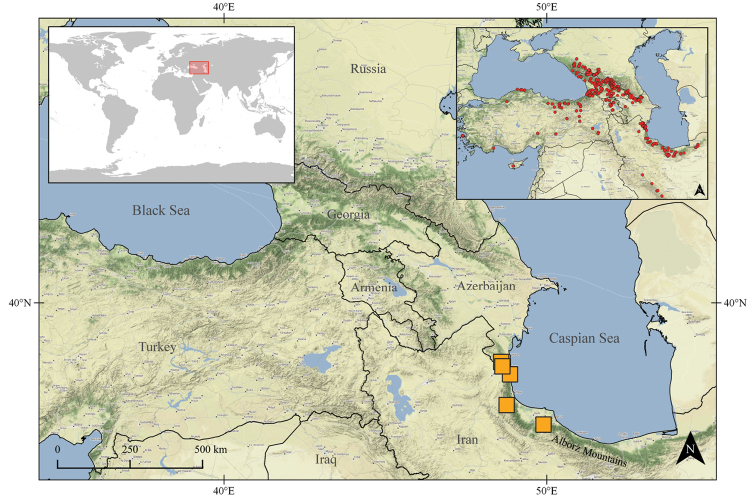
Location of study area (left corner), overall sampling (right corner), and distribution of Epeorus (Caucasiron) hyrcanicus, sp. nov. (orange squares).

This species is distributed in the Hyrcanian forest of southeastern Azerbaijan and northwestern Iran (Fig. 1). The forest stretches along the southern shores of the Caspian Sea in Azerbaijan and Iran (Soofi et al. 2018) and covers the lowlands and foothills of the northern slopes of the Alborz Mountains (Gholizadeh H et al. 2019). It has a high conservation value and represents a tertiary relict temperate forest (Soofi et al. 2018).

Our extensive sampling in the Caucasus and surrounding areas (Fig. 1) points to a relatively narrow distribution of the new species, which is likely restricted to the Hyrcanian forest. It may represent a species endemic to this area, similar to E. (C.) iranicus, E. (C.) alborzicus, and E. (C.) shargi (Hrivniak et al. 2020a, 2020b).

The new species was found to be related to E. (C.) caucasicus, E. (C.) nigripilosus, and E. (C.) turcicus, and its origin is dated to the Pliocene (Hrivniak et al. 2020c). During this period, intensive orogenic activity and climate cooling took place in the Caucasus region, causing fragmentation of ancient forests to isolated patches (Popov CV et al. 2004; Naderi et al. 2014; Tarkhnishvili 2014). The Hyrcanian forest, together with the Pontic Mountains and Colchis lowland, was an important forest refugium where forest-associated species found suitable and stable environmental conditions during this period (cf. Tarkhnishvili 2014). This allowed the survival of tertiary relicts (Erichsen EO et al. 2017), resulting in a high number of local endemics (Naderi et al. 2014). We assume that these historical events could affect the evolutionary diversification of lineages in *Caucasiron* distributed along the southern Caspian Sea coastal areas.

Although the discovery of distinct lineages represents an essential step for evolutionary studies, their morphological determination is required for practical purposes of nature conservation and biomonitoring surveys. Thus, we aim to complete our phylogenetic studies with morphological investigations and taxonomy of evolutionary lineages delimited by molecular data.

The main aims of this study are to: i) investigate larval morphology of the lineage *Caucasiron* sp. 3 delimited as a putative species by Hrivniak et al. (2020c) and provide morphological comparison with other Caucasian *Caucasiron* species, ii) apply molecular species delimitation methods to our COI dataset containing all currently described Caucasian *Caucasiron* species, and iii) provide diagnostic characters for identification of the new species together with basic information on its habitat requirements.

## Material and methods

The material used for this study was collected by J. Bojková, T. Soldán, and J. Imanpour Namin in Iran (May 2016), and Ľ. Hrivniak, P. Manko, D. Murányi, and M. Žiak in Azerbaijan (September 2018). Larvae were collected by hand net and preserved in 75–96% EtOH. Other *Caucasiron* species, used for morphological comparisons and molecular analyses, were obtained from the collections of the Biology Centre of the Czech Academy of Sciences, Institute of Entomology, České Budějovice, Czech Republic (IECA).

### Morphological examination

Parts of larval specimens were mounted on microscopic slides using HydroMatrix (MicroTech Lab, Graz, Austria) mounting medium. In order to remove the muscle tissue for an investigation of the cuticular structures, specimens were left overnight in a 10% solution of NaOH prior to slide mounting. Drawings were made using an Olympus SZX7 stereo microscope and an Olympus BX41 microscope, both equipped with a drawing tube. Photographs were obtained using a Leica DFC450 camera on a Leica Z16 APO macroscope and stacked in Helicon Focus v. 5.3 X64. All photographs were subsequently enhanced with Adobe Photoshop CS5. Diagnostic characters for the description of larva were chosen according to Hrivniak et al. (2020a).

### DNA extraction, PCR, sequencing, and alignment

Total genomic DNA of the species was extracted from legs using the DEP-25 DNA Extraction Kit (TopBio s.r.o., Prague, Czech Republic) according to the manufacturer’s protocol. Mitochondrial cytochrome oxidase subunit I (COI) was sequenced according to Hrivniak et al. (2017). COI sequences were obtained from seven specimens (three from Iran and four from Azerbaijan). COI sequences of other *Caucasiron* species were obtained from Hrivniak et al. (2017) (GenBank accession numbers (GB): KY865691–KY865725), Hrivniak et al. (2019) (GB: KY865691–KY865725), and Hrivniak et al. (2020b) (GB: MN856180–MN856198). The PCR amplification of COI and reaction volumes was carried out as described in Hrivniak et al. (2017). Sequences were assembled in Geneious v. 7.0.6 (http://www.geneious.com) and aligned in the same software using the Mafft v. 7.017 (Katoh et al. 2002) plugin with default settings. Newly obtained sequences are deposited in GenBank with accession numbers MZ389776–MZ389782.

### Molecular species delimitation

Species were delimited using the single locus (COI) coalescence based General Mixed Yule Coalescent model (GMYC, Pons et al. 2006; Fujisawa and Barraclough 2013). We applied the single-threshold GMYC model as it has been found to outperform the multi-threshold (Fujisawa and Barraclough 2013) and was found to be highly suitable for species delimitation within *Caucasiron* (Hrivniak et al. 2019, 2020b).

Analyses were performed using the SPLITS package for R (http://r-forge.rproject.org/projects/splits). An ultrametric COI gene tree was reconstructed in BEAST 2 (Bouckaert et al. 2014) with the settings as described in Hrivniak et al. (2020b).

Inter- and intra-specific uncorrected pairwise genetic distances were calculated in MEGA X (Kumar et al. 2018). Distance-based species delimitation was performed using Automatic Barcode Gap Discovery (ABGD) (Puillandre et al. 2012) (online version: http://wwwabi.snv.jussieu.fr/public/abgd/) with default settings.

## Results and discussion

### Epeorus (Caucasiron) hyrcanicus

Taxon classificationAnimaliaEphemeropteraHeptageniidae

Hrivniak & Sroka sp. nov.

3CAFD9C5-F391-5B42-9029-14E2B529FA07

http://zoobank.org/070D9D7B-CDE6-42D2-A3EB-2B77665C3C55

Figures 4 , 5

Caucasiron sp. 3 of Hrivniak et al. 2020c

#### Notes.

Epeorus (Caucasiron) hyrcanicus sp. nov. is attributed to the subgenusCaucasiron within the genus *Epeorus* s.l. based on the following larval morphological characters: i) projections on the costal rib of gill plates II–VII, ii) presence of medio-dorsally directed hair-like setae located on the anterior margin of the head (see Kluge 2015 for a revision of the subgenusCaucasiron).

**Figure 2.**
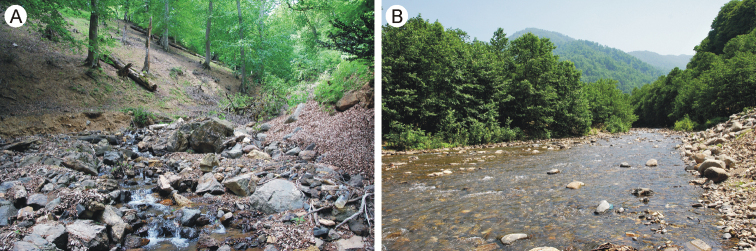
Localities of Epeorus (Caucasiron) hyrcanicus sp. nov. in Iran **A** unnamed brook near Sangdeh village (locality no. 12) **B** Shamrud River near Tushi village (locality no. 17).

#### Type material.

***Holotype***: female larva: Azerbaijan, Lənkəran Province, NW of Azaru village, unnamed brook (left tributary (LT) of Vasharu River); 38.5873689N, 48.5870392E (locality AZE5/2018); 1028 m a.s.l.; Ľ. Hrivniak, M. Žiak leg., 21.9.2018. ***Paratypes***: 5 larvae (2 barcoded and mounted on slide; labelled CFC1, CFC2): same data as holotype.

**Figure 3.**
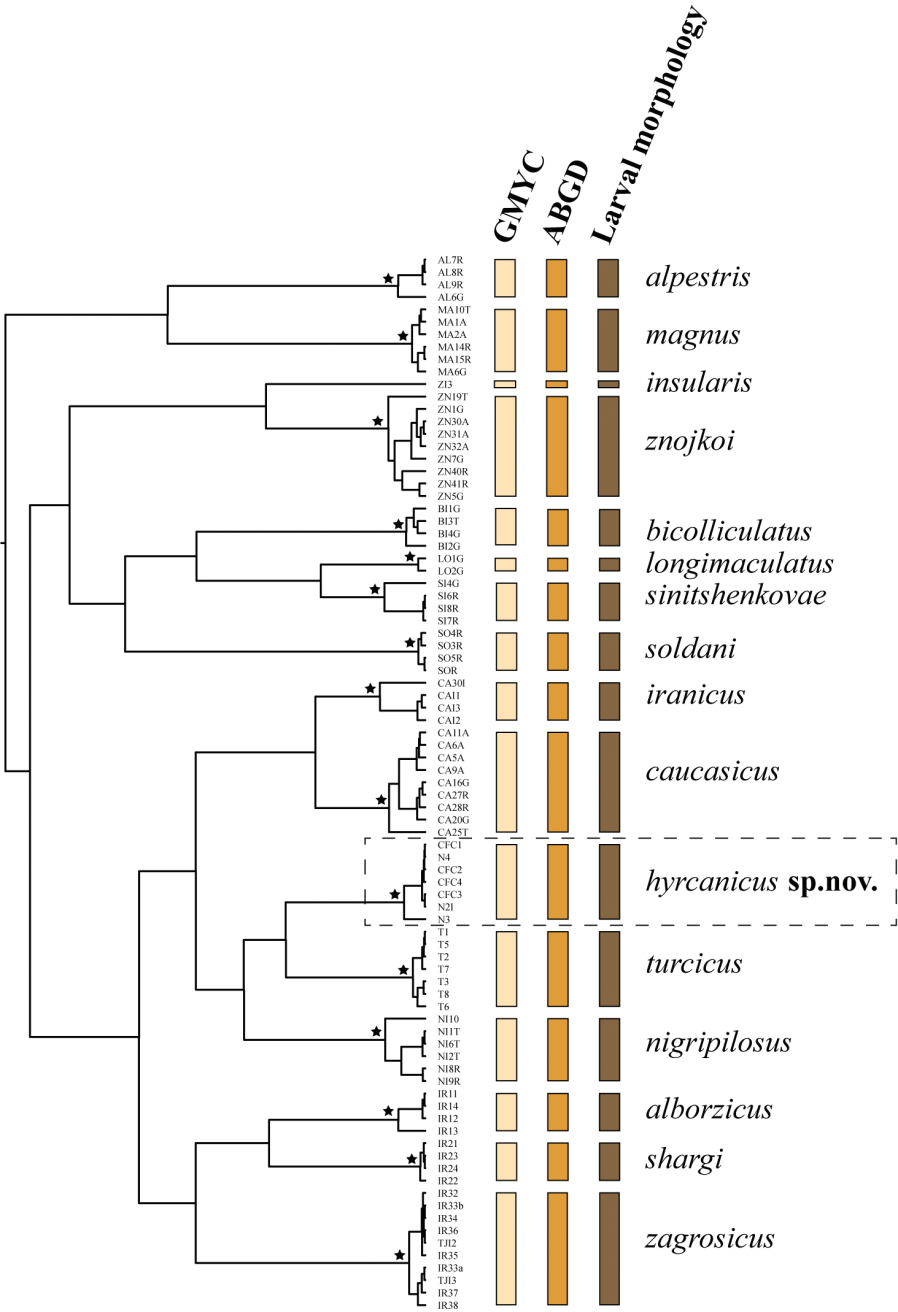
COI gene tree with the results from molecular species delimitation and larval morphology. Stars near nodes represent posterior probability 1. Delimitation of Epeorus (Caucasiron) hyrcanicus, sp. nov. highlighted by dashed rectangle.

1 larva (mounted on slide): Azerbaijan, Lənkəran Province, SW of Sim village, unnamed brook (tributary of Digo River); 38.4824842N, 48.6243081E (locality AZE6/2018); 734 m a.s.l.; Ľ. Hrivniak, P. Manko leg., 21.9.2018.

4 larvae (one barcoded and mounted on slide; labelled N4): Iran: Gilan Province, NW of Sangdeh village, unnamed brook (LT of Shafa-rud River); 37.5294444N, 48.7552778 E (locality no. 12); 1345 m a.s.l.; J. Bojková, T. Soldán, J. Imanpour Namin leg., 15.5.2016.

1 larva (barcoded; labelled N2): Iran, Gilan Province, S of Tushi village (S of Siahkal village), Shamrud River (RT of Sefid-rud River); 37.0500000N, 49.8983333E (locality no. 17); 314 m a.s.l.; J. Bojková, T. Soldán, J. Imanpour Namin leg., 16.5.2016.

#### Other material (damaged larvae).

4 larvae (2 barcoded and mounted on slide; labelled CFC4, CFC3): same data as holotype.

1 larva (barcoded; labelled N3): Iran, Gilan Province, W Chelvand village (S of Lavandvil village), Chelavand River (about 2.5 km from its mouth); 38.2888889N, 48.8597222E (locality no. 27); -4 m a.s.l.; J. Bojková, T. Soldán, J. Imanpour Namin leg., 19.5.2016.

#### Etymology.

The name refers to the distribution of the species in the Hyrcanian forest.

#### Distribution and habitat preferences of larvae.

The species is distributed in northwestern Iran and southeastern Azerbaijan (Fig. 1) at -4 to 1345 m a.s.l. Larvae were found in streams and rivers flowing to the Caspian Sea in the humid forested slopes of western Alborz. They likely inhabit only cold and clear streams and rivers with stony bed substrate and turbulent flow. The species was not found in urban and agricultural areas in this region where many localities were investigated. Larvae were not abundant in either locality and co-occurred with the more abundant E. (C.) znojkoi.

#### Description of larva.

General coloration of larvae yellowish-brown, with dark brown to reddish maculation. Body length of male mature larva 8.25 mm (*n* = 1); cerci broken. Body length of female mature larvae unknown.

**Figure 4.**
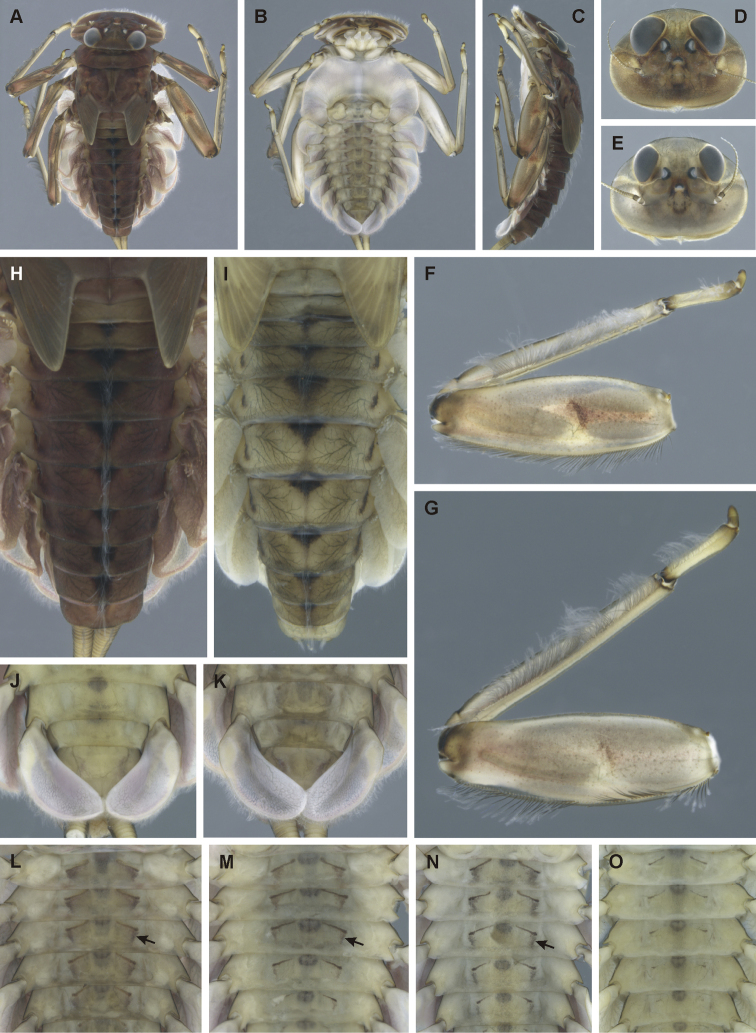
Epeorus (Caucasiron) hyrcanicus, sp. nov., larva **A** habitus in dorsal view **B** habitus in ventral view **C** habitus in lateral view **D** head of male in dorsal view **E** head of female in dorsal view **F, G** middle leg in dorsal view **H, I** abdominal terga **J, K** gills VII (in natural position from ventral view) **L–O** abdominal sterna II–VI (arrow points on medio-lateral maculae).

**Head.** Shape oval to trapezoidal (Fig. 4D, E). Anterior margin with shallow concavity medially. Head dimensions: length 2.20 mm, width 3.04 mm (male); dimensions of female mature larva unknown. Head width/length ratio 1.36–1.41 (male), 1.40–1.44 (female). Coloration pattern as in Figure 4D, E. Dorsal surface of head covered with fine hair-like setae and sparsely distributed stick-like setae. Sparse longer hair-like setae located posteriorly to eyes.

**Figure 5.**
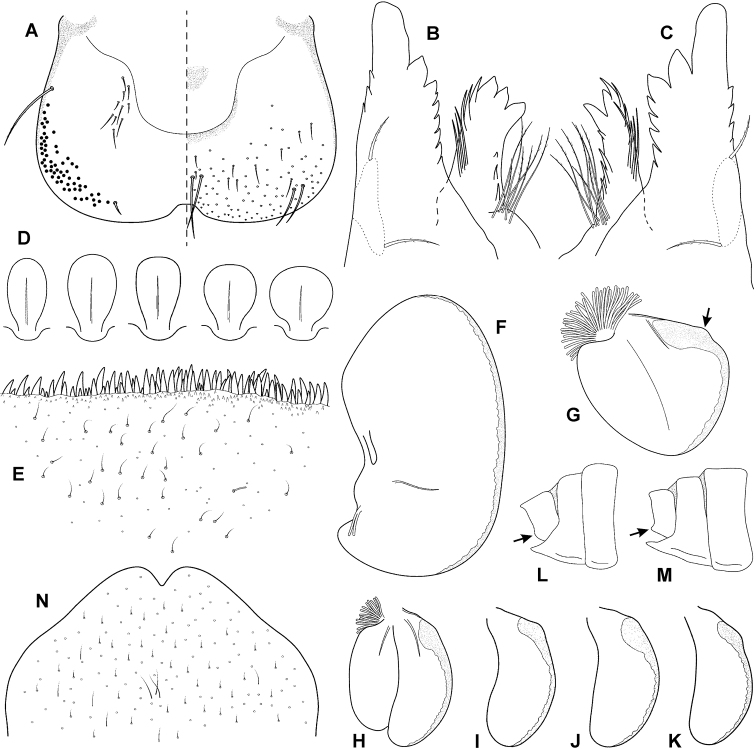
Epeorus (Caucasiron) hyrcanicus, sp. nov., larva **A** labrum (left half in ventral view, right half in dorsal view) **B** incisors of left mandible **C** incisors of right mandible **D** setae on dorsal surface of femora **E** surface and posterior margin of abdominal tergum VII **F** gill I **G** gill III **H** gill VII (flattened on slide) **I–K** gill VII (in natural position from ventral view) **L, M** abdominal segments VIII–X in lateral view (arrow points on postero-lateral projection) **N** sternum IX of female.

**Mouthparts.** Labrum (Fig. 5A) widened anteriorly, with anterior margin slightly rounded (in dorsal view). Lateral angles rounded (shape of labrum may vary among specimens). Dorsal surface (Fig. 5A, right half) sparsely covered with setae of different size; four longer bristle-like setae located antero-medially and two antero-laterally. Epipharynx with longer, slightly plumose bristles situated along lateral to anterior margin (Fig. 5A, left half; range of setation figured as large black dots), and cluster of fine, hair-like setae medially (not figured). Posterior margin of labrum irregularly concave; group of 11–13 setae of various sizes located on ventral surface close to posterior margin. Outer incisors of both mandibles (Fig. 5B, C) with three apical teeth; outer tooth blunt in both mandibles. Inner incisor of left mandible with three apical teeth (Fig. 5B), right inner incisor bifurcated (Fig. 5C).

***Thorax*.** Pronotum anteriorly narrowed, lateral edges nearly straight. Metanotum with slight postero-medial projection. Dorsal surface covered with fine, hair-like setae (as on abdominal terga and head); sparse longer hair-like setae along pro-, meso- and metanotal suture.

***Legs*.** Colour pattern of femora as in Figure 4F, G. Femora with medial hypodermal spot; often distally blurred (Fig. 4F) or poorly expressed (Fig. 4G). Patella-tibial suture darkened; tarsi proximally and distally darkened. Coxal projections of fore- and hind legs pointed or bluntly pointed; in middle legs blunt. Spatulate setae on dorsal surface of femora short, sporadically elongated (Fig. 5D). Tarsal claws with 3–4 denticles.

***Abdominal terga*.** Colour pattern of abdominal terga (Fig. 4A, H–I) consisting of transversal stripe along anterior margin of terga I–IX (X), medially extending, forming rectangular or triangular macula on terga II–IV (sometimes blurred), triangular macula on terga V–VII, and triangular or rectangular macula on terga VIII–IX (X). Lateral margins with oblique maculae on terga I–VIII (IX). Denticles on posterior margin of terga relatively dense, of various sizes, pointed and sometimes curved (Fig. 5E). Surface of terga covered with hair-like setae and sparsely with stick-like setae. Tergum X with short or without postero-lateral projections (Fig. 5L, M; arrow). Medial longitudinal row of hair-like setae along abdominal terga present.

***Abdominal sterna*.** Yellowish, with dark brown to blackish pattern. Sterna II–VI with a pair of oblique stripes (medio-anterior sigilla) and a pair of stripe-like (or elongated triangular) medio-lateral maculae joined to medio-anterior sigilla (Fig. 4L–N; arrows). Intensity of colouration varies among individuals; stripe-like (or elongated triangular) medio-lateral stripes sometimes poorly developed (Fig. 4O). Nerve ganglia darkened. Sternum IX with V-shaped medial emargination; surface covered by irregularly distributed hair-like setae (Fig. 5N).

***Gills*.** Dorsal surface of gill plates I yellowish; of gill plates II–VII yellowish on anterior half, brownish to reddish on posterior half. Ventral margin of all gill plates yellowish to greyish. Projection of gill plates III poorly developed (Fig. 5G; arrow). Gill plates VII relatively wide (in natural position of ventral view; Fig. 4J, K; 5H–K).

***Cerci*.** Yellowish brown, basally darkened.

#### Subimago, imago and eggs.

Unknown.

#### Morphological diagnostics of larvae.

The main larval diagnostic characters of E. (C.) hyrcanicus sp. nov. are as follows: (i) abdominal sterna II–VI with pair of oblique stripes (Fig. 4L–O) and stripe-like (or elongated triangular) medio-lateral maculae (Fig. 4L–N; arrows), (ii) terga V–VII with triangular medial maculae (Fig. 4H, I), iii) femora with medial hypodermal spot, sporadically absent, reduced, or distally blurred (Fig. 4F, G), (vi) fine hair-like setae on surface of abdominal terga (Fig. 5E), (v) poorly developed projection on gill plates III (Fig. 5G; arrow), and (vi) relatively wide shape of gill plates VII (in natural position from ventral view; Fig. 4J, K; 5H–K).

#### Results from molecular species delimitation.

The GMYC species delimitation model applied to COI gene tree provided significantly better fit for a speciation branching than null model suggesting uniform coalescent branching across the entire tree (likelihood ratio test = 6.258895e-07***). The GMYC estimated 16 species (CI = 11–18) consisting of 15 ML clusters and one singleton (E. (C.) insularis). Morphologically defined E. (C.) hyrcanicus sp. nov. was delimited as a distinct species based on both GMYC and ABGD species molecular delimitation analyses. All species clusters were highly supported (PP = 1; Fig. 3).

The intraspecific pairwise genetic distances between specimens of E. (C.) hyrcanicus sp. nov. reached up to 1.75%. The minimum (and mean) interspecific distances between E. (C.) hyrcanicus sp. nov. and other *Caucasiron* species ranged between 8.11% (8.84%; E. (C.) nigripilosus) and 15.52% (15.88%; E. (C.) alpestris).

#### Morphological affinities.

Although the combination of larval morphological diagnostic characters listed above clearly determine E. (C.) hyrcanicus sp. nov. from all *Caucasiron* species known so far, some of the species distributed in the Caucasus and adjacent areas possess nearly identical states of some characters. Distinguishing of E. (C.) hyrcanicus sp. nov. from these species is described in detail below.

Coloration pattern of abdominal sterna II–VI makes E. (C.) hyrcanicus sp. nov. the most similar to E. (C.) caucasicus (widely distributed in the Caucasus), E. (C.) iranicus (distributed in Alborz Mountains), and E. (C.) zagrosicus (distributed in Zagros Mountains). The pattern of all these species consists of a pair of oblique stripes (medio-anterior sigilla). E. (C.) hyrcanicus sp. nov. usually exhibits oblique stripes together with a pair of stripe-like (or elongated triangular) medio-lateral maculae (Fig. 4L–N; arrows), in contrast to E. (C.) caucasicus and E. (C.) iranicus without such maculation (Hrivniak et al. 2020a: figs 4J, 22I, J).

Weakly pigmented specimens of E. (C.) hyrcanicus sp. nov., i.e. without distinctly pigmented medio-lateral maculae on abdominal sterna II–VI (Fig. 4O) possess the same coloration pattern as in E. (C.) caucasicus and E. (C.) iranicus. Such specimens are identifiable by the triangular shape of medial maculae on abdominal terga V–VII (Fig. 4H, I). In contrast to E. (C.) hyrcanicus sp. nov., E. (C.) caucasicus bears crown-like medial maculae on terga V–VII (Hrivniak et al. 2020a: fig. 4I), and E. (C.) iranicus stripe-like medial maculae with distinct antero-lateral stripes (Hrivniak et al. 2020a: fig. 22G).

The oblique stripes on abdominal sterna II–VI in E. (C.) zagrosicus are anteriorly widened (Hrivniak et al. 2020a: fig. 46I). This feature separates this species from E. (C.) hyrcanicus sp. nov.

In E. (C.) hyrcanicus sp. nov., the coloration pattern of abdominal terga V–VII, legs, and shape of gill plates VII (in natural position from ventral view) is similar to E. (C.) shargi (distributed in the eastern Alborz Mountains) (Hrivniak et al. 2020a: fig. 43). From this species, E. (C.) hyrcanicus can be distinguished by the presence of the coloration pattern on abdominal sterna II–VI (Fig. 4L–O), in contrast to E. (C.) shargi, where the pattern is missing (Hrivniak et al. 2020a: fig. 43L). The poorly developed projection on gill plates III in E. (C.) hyrcanicus sp. nov. (Fig. 5G; arrow) also differs from E. (C.) shargi with well-developed projection (Hrivniak et al. 2020a: figs 44G). Additionally, E. (C.) hyrcanicus usually bears short postero-lateral projection on tergum X (Fig. 5M; arrow), in contrast to E. (C.) shargi without such projection (Hrivniak et al. 2020a: fig. 44J).

Well-defined triangular medial maculae on abdominal terga V–VII are characteristic also for E. (C.) soldani (distributed in the western and central Greater Caucasus Mountains). Epeorus (C.) hyrcanicus sp. nov. can be separated from E. (C.) soldani by hair-like setae on abdominal terga (Fig. 5E), in contrast to the wide setae in E. (C.) soldani (Hrivniak et al. 2020a: fig. 20E). Additionally, the gill plates VII (in natural position from ventral view) are wider in E. (C.) hyrcanicus sp. nov., in contrast to E. (C.) soldani with narrow shape (Hrivniak et al. 2020a: fig 19L), and the projection on gill plates III is poorly developed in E. (C.) hyrcanicus sp. nov., in contrast to E. (C.) soldani with well-developed projection (Hrivniak et al. 2020a: fig. 20G).

## Supplementary Material

XML Treatment for Epeorus (Caucasiron) hyrcanicus
